# Intestinal parasitosis in school children of Lalitpur district of Nepal

**DOI:** 10.1186/1756-0500-6-449

**Published:** 2013-11-09

**Authors:** Sarmila Tandukar, Shamshul Ansari, Nabaraj Adhikari, Anisha Shrestha, Jyotshana Gautam, Binita Sharma, Deepak Rajbhandari, Shikshya Gautam, Hari Prasad Nepal, Jeevan B Sherchand

**Affiliations:** 1 Public Health Research Laboratory, Institute of Medicine, Tribhuvan University Teaching Hospital, Kathmandu, Nepal; 2 Department of Microbiology, Chitwan Medical College, Bharatpur, Chitwan, Nepal; 3 Kantipur College of Medical Science, Sitapaila, Kathmandu, Nepal; 4 Kirtipur Hospital, Kathmandu, Nepal

**Keywords:** *Giardia lamblia*, Intestinal parasite, Nepal, School children

## Abstract

**Background:**

Enteric parasites are the most common cause of parasitic diseases and cause significant morbidity and mortality, particularly in developing countries like Nepal. The objective of this study was to estimate the prevalence and risk factors of intestinal parasitic infections among school going children of Lalitpur district of Nepal.

**Methods:**

A total of 1392 stool samples were collected from school children of two government, two private and two community schools of the same district. The stool samples were examined for evidence of parasitic infections by direct microscopy and confirmed by concentration methods (formal ether sedimentation technique or floatation technique by using Sheather’s sugar solution). Modified Ziehl-Neelsen (ZN) staining was performed for the detection of coccidian parasites.

**Results:**

Prevalence of intestinal parasitosis was found to be 16.7%. The highest prevalence rate was seen with *Giardia lamblia* (7.4%) followed by *Entamoeba histolytica* (3.4%) and *Cyclospora cayetanensis* (1.6%). Children aged 11–15 years and the ones belonging to family of agriculture workers were most commonly affected. Hand washing practice and type of drinking water also showed significant difference.

**Conclusions:**

The burden of parasitic infections among the school children, coupled with the poor sanitary conditions in the schools, should be regarded as an issue of public health priority and demands for effective school health programs involving periodic health education and screening.

## Background

Intestinal parasitic infection is a serious public health problem throughout the world particularly in developing countries [1]. It was estimated to affect around 3.5 billion people globally and 450 million people were ill due to parasitic infection [2]. It continues to be a major cause of morbidity and mortality and was most common in school-going children, street children, farmers and their children due to use of contaminated drinking water, inadequate sanitary conditions and poor personal hygiene [3-9]. A study conducted in two rural villages of Chitwan, Nepal in 1999 showed a 44% prevalence of intestinal parasitosis in school-going children [9], whereas a similar study from Pokhara, Nepal in 2004 showed a lower prevalence of 21.3% [2].

In Nepal, over 70% of morbidity and mortality are associated with infectious diseases and is also reflected in the “top ten diseases” of Nepal [10]. Diarrhea is produced by a variety of etiological agents. Of them, intestinal parasitic infection alone contributes to a great extent in the cause of diarrhea and is one of the most common public health problems in Nepal [11]. The common intestinal helminths reported from Nepalese children are *Ascaris lumbricoides*[10,12], hookworm, and *Trichuris trichiura*[13], with manifestations as varied as malnutrition, iron deficiency anemia, mal-absorption syndrome, intestinal obstruction, and mental and physical growth retardation. Of the protozoal infections, amoebiasis and giardiasis are most frequently reported. The agents spread faeco-orally through contaminated sources. Although people of all ages may be infected by these organisms, children are more often infected due to compromise in sanitary habits [14]. Therefore the objective of this study was to determine the prevalence and risk factors of intestinal parasitosis among school children. The findings of this study might help in strengthening the information available so far and encourage policy makers to design effective strategies to prevent intestinal parasitic infections in the study area.

## Methods

### Study type and area

This was a school based cross-sectional study conducted from July 2011 to December 2011. The study population comprised school going children upto 15 years of age. The study area was the Lalitpur district of Nepal, a landlocked country of South Asia. The district, bordering Kathmandu (capital city of Nepal) in its northern part, covers an area of 385 km^2^ and has a total population of 468132 (2011). Two schools each from all the three categories- government, private and community schools located in this district were selected to include the children from various socio-economic standards.

### Sample collection

A total of 1392 children upto the age of 15 years, who did not have any disabilities and were not under any anti-parasitic medication, were enrolled in this study. The study population was divided into 3 age groups, i.e. upto 5 years, 6–10 years and 11–15 years. The data were collected by well trained medical personnel. A short questionnaire was designed which included **a) socio-demographic data:** address, age, gender, socio-economic status **b) behavioral data:** hand washing habits and types of drinking water **c) participant’s present medical history:** any complaints of abdominal pain/discomfort, nausea and vomiting. Children were interviewed in their mother tongue. All the questionnaires were checked for accuracy and completeness. After proper instructions were given to the children regarding collection of the stool sample, they were given labeled collection-containers and application sticks. From each student, about 2 g of fresh stool was collected. Each of the specimens were checked for its labeling and quantity. A portion of the stool samples was processed immediately to detect cysts, trophozoites, eggs and larva of intestinal parasites. The remaining part was preserved in 10% formalin solution and transported to the laboratory for further investigation following the standard laboratory protocol (WHO protocol) in the Public Health Research Laboratory, Institute of Medicine, Tribhuvan University Teaching Hospital (TUTH), Kathmandu, Nepal.

### Macroscopic examination

The colour, consistency, presence of blood and mucus and any other abnormalities were observed macroscopically.

### Microscopic examination

Microscopic examination of all the stool specimens were carried out by wet preparation (normal saline and iodine), concentration and modified Ziehl-Neelsen (ZN) methods. Direct wet mounts of stool specimens were prepared in a drop of normal saline for the observation of pus cells, ova, cyst and trophozoites of parasites. Iodine preparations of stools were prepared in 5 times diluted Lugol’s iodine. Concentration methods (formal-ether sedimentation technique or floatation technique by using Sheather’s sugar solution) and modified ZN staining technique [15] were employed for all the stool specimens. Special techniques for the *Enterobius vermicularis* and *Strongyloides stercoralis* were not performed in this study.

### Ethical consideration

This study was approved by the Institutional Review Committee of Chitwan Medical College (Reference: CMC-IRC-32), Bharatpur, Nepal. Informed written consent was taken from the head of participating school and parents of all the participating children.

### Data analysis

Statistical analysis was performed using Epi-Info and SPSS-11.5 version. Association of intestinal parasitosis with demography, personal habits and symptoms were assessed by using the Chi-square test. P values < 0.05 were considered as statistically significant.

## Results

### Prevalence of parasites

Among a total of 1392 children belonging to six schools enrolled in the study, 732 were male and 660 were female (male to female ratio of 1.1:1). The overall prevalence rate of the parasitic infection in those children was found to be 16.7% (male-17.8% and female-15.5%). Of 232 positive cases, protozoal parasites were found in 81.5% cases while helminthic parasites in 18.5% of cases. The prevalence of protozoal and helminthic infections were found to be 13.6% and 3.1% respectively.

The highest rate of intestinal parasitosis was seen in the age group of 11–15 years and in the children belonging to government school (Tables 1 and 2).

**Table 1:** Age wise distribution of different entero-parasites

**Frequency of intestinal parasites**	**Upto 5 years**	**6-10 years**	**11-15 years**	**Total**	**Prevalence (%)**
	**(Total cases = 464)**	**(Total cases = 464)**	**(Total cases = 464)**		
**Protozoa**	**35**	**73**	**81**	**189**	**13.6**
*Giardia lamblia*	20	42	41	103	7.4
*Entamoeba histolytica*	6	14	27	47	3.4
*Cyclospora cayetanensis*	6	10	7	23	1.6
*Entamoeba coli*	3	6	4	13	1.0
*Blastocystis hominis*	0	1	2	3	0.2
**Helminths**	**6**	**16**	**21**	**43**	**3.1**
*Hymenolepis nana*	0	3	10	13	1.0
*Ascaris lumbricoides*	2	2	6	10	0.7
*Trichuris trichiura*	0	4	1	5	0.4
*Enterobius vermicularis*	2	3	1	3	0.4
*Taenia spp.*	0	1	2	3	0.2
*Strongyloides stercoralis*	1	2	0	3	0.2
*Ancylostoma duodenale*	1	1	1	3	0.2
**Total**	**41 (8.83%)**	**89 (19.18%)**	**102 (21.98%)**	**232**	**16.7**

**Table 2:** Distribution of entero-parasites in different types of school

**Intestinal parasites**	**Private school**	**Government**	**Community**	**Total**	**Prevalence (%)**
	**(Total cases = 450)**	**School (Total cases = 475)**	**School (Total cases = 467)**		
**Protozoa**	**41**	**133**	**15**	**189**	**13.6**
*Giardia lamblia*	27	69	7	103	7.4
*Entamoeba histolytica*	8	37	2	47	3.4
*Cyclospora cayetanensis*	4	16	3	23	1.6
*Entamoeba coli*	2	9	2	13	1.0
*Blastocystis hominis*	0	2	1	3	0.2
**Helminths**	**3**	**37**	**3**	**43**	**3.1**
*Hymenolepis nana*	0	13	0	13	1.0
*Ascaris lumbricoides*	2	6	2	10	0.7
*Trichuris trichiura*	0	5	0	5	0.4
*Enterobius vermicularis*	0	6	0	3	0.2
*Taenia spp.*	0	3	0	3	0.2
*Strongyloides stercoralis*	0	3	0	3	0.2
*Ancylostoma duodenale*	1	1	1	3	0.2
**Total**	**44 (9.8%)**	**170 (35.8%)**	**18 (3.9%)**	**232**	**16.7**

**Figure 1:**
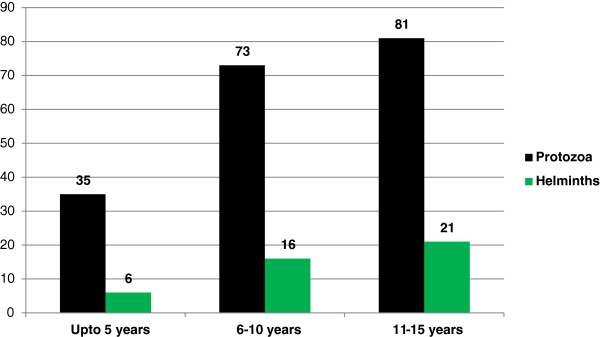
Distribution of Protozoal and Helminthic enteroparasites in different age groups.

**Figure 2:**
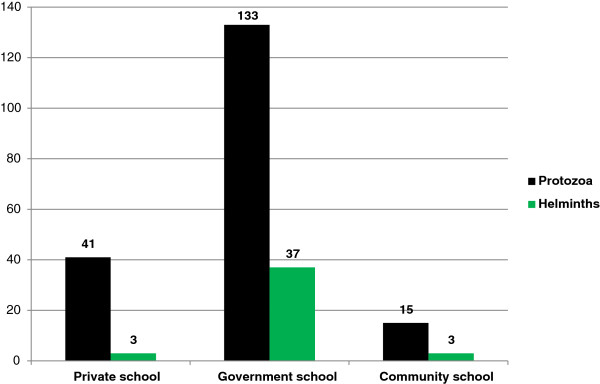
Distribution of Protozoal and Helminthic enteroparasites in different types of schools.

Among 189 protozoal parasites positive cases, the highest number of cases belonged to age group of 11-15 years (42.8%) and the children of government school (70.4%). Similarly, of 43 helminthic parasites positive cases, majority of cases belonged to age group of 11-15 years (48.8%) and the children of government school (86.0%) as shown in Figures 1 and 2. Mixed infections were also found in 23 cases (1 case of helminth + helminth, 6 cases of helminth + protozoa and 16 cases of protozoa + protozoa). Of all the intestinal parasites detected, the most common parasite was *Giardia lamblia* (44.4%) followed by *Entamoeba histolytica* (20.26%) with prevalence of *Giardia lamblia* to be 7.4% followed by *Entamoeba histolytica* to be 3.4% (Tables 1 and 2).

### Possible risk factors and associated medical history

The children whose major family occupation was agriculture had significantly high positivity rate of intestinal parasitosis (24.3%) compared to others (P < 0.05) as shown in Table 3.

**Table 3:** Possible risk factors and associated medical history of parasites positive children

**Variables**	**Total cases, N = 1392**	**Positive cases, N = 232 (%)**	**X**^**2**^**-Value**	**P-Value**
**Gender**			1.32	0.21
Male	732	130 (17.8)		
Female	660	102 (15.5)		
**Occupation**			28.63	0.000238
Agriculture	584	142 (24.3)		
Business	375	48 (12.8)		
Services	225	20 (8.9)		
Abroad	113	12 (10.6)		
Others	95	10 (10.5)		
**Hand washing practice**			213.01	0.000013
Found	1137	111 (9.8%)		
Not found	255	121 (47.5%)		
**Type of drinking water**			96.69	0.000029
Boiled tap water	871	79 (9.0%)		
Direct tap water	521	153 (29.4%)		
**Gastrointestinal symptoms**			44.60	0.000062
Not found	515	41 (8%)		
Found	877	191 (21.8%)		

Intestinal parasitosis was significantly associated with gastrointestinal symptoms as depicted in Table 3.

Intestinal parasitosis was found more common in those children who did not follow hand washing practice (47.5%) and used direct tap (unboiled) water for drinking purpose (29.4%) in comparison to others (P < 0.05).

## Discussions

Intestinal parasitic infections are among the most common infections worldwide. Its prevalence depends upon various socio-economic factors such as hygiene, availability of clean drinking water, poverty, etc. Prevalence rate of intestinal parasitosis observed in this study (16.7%) concords with the rates reported by various authors from other parts of Nepal [2, 16-18]. Slightly higher infection rate in male over female seen in the present study was statistically non-significant (P = 0.21).

Protozoal parasites were found more common (13.6) than the helminthic parasites (3.1%) in the current study. Protozoa dominating the helminthic parasites is in agreement with the previous findings from Nepal [8,13]. Among protozoal parasites, *Giardia lamblia* is the most common flagellate of the intestinal tract, causing giardiasis in humans and are the only important reservoir of the infection, and infection is most common in parts of the world where sanitation is at its lowest. *Entamoeba histolytica* is the only species found to be associated with intestinal disease. Although many people harbor this organism worldwide, only about 10% develop clinically invasive disease. Giardiasis is an infection of the upper small bowel, which may cause diarrhea. In our study, the most frequently seen protozoal parasite was *Giardia lamblia* (7.4%) followed by *Entamoeba histolytica* (3.4%). Higher prevalence of *Giardia lamblia* (13.2%) and *Entamoeba histolytica* (1.7%) was also reported by Chandrashekhar et al. [2]. Higher prevalence of *Giardia lamblia* followed by *Entamoeba histolytica* was also detected from other parts of Nepal [16,19]. It indicates the need of protection from fecal contamination in the locality and correction of water supply system. Of the protozoal infections, and giardiasis are the most frequently reported infections which spread by faeco-orally through contaminated sources. The by faeco-orally through contaminated sources. School-age children are particularly susceptible to parasitosis, often carrying higher burdens of parasites than adults [20]. The greatest obstacle to effective control of parasites in at-risk populations is inadequate knowledge of the geographical distribution of infection and the demographic variables that influence the prevalence of infection [20].

In this study, *Hymenolepis nana* was the most common helminth detected which is a noteworthy finding similar to the observations of Sharma et al., Adhikari et al. and Shrestha et al. [7,21,22]. Higher prevalence of hymenolepiasis was also reported by Wadood et al., from Pakistan, Martinez et al., from Mexico and by Mirdha et al., from India [23-25]. The *Ascaris lumbricoides* as the common helminth among school children have been reported by several other authors [5,11,16,26]. In our study helminthic infections were less prevalent as compared to the protozoal infections, although studies from other parts of Nepal have shown a higher prevalence of soil transmitted helminths [2,3,5,6,9]. Helminthic infections are associated with nutritional deficiencies, particularly of iron and vitamin A, with improvements in iron status and increments in vitamin-A absorption seen after deworming [18]. Thus, periodic campaign of anti-helminthic drug administration by ministry of health to the children could possibly explain the lower prevalence of helminthic infections seen in this study.

High prevalence rate (7.3%) of intestinal parasitosis was found in children aged 11–15 years, the findings being similar to the observation of Shrestha et al. [18]. Most children of this age group are fascinated towards street food and drinks which may be important predisposing factors for high prevalence of parasitic infection in this age group. Among the three categories of schools, intestinal parasites were found more common in the children of government school. The government schools have the students primarily from low- income families while the private schools from high-income families and the community schools from middle-income families. Therefore, our findings highlight the link between prevalence of intestinal parasitic infection and the economic status of the family which the children belongs to [16,19].

In addition, the significant association between the prevalence of intestinal parasitic infection and the occupational status of the family was found. Agriculture as a major family occupation was found as the most predisposing factor for the intestinal parasitosis (24.3%) whereas the least parasites positive cases (8.9%) were seen in the children whose major family occupation was services. Our result is in accordance with the report of Shrestha et al. [18]. This may be due to the fact that the children and other members of this occupational family work in the field and they transmit the parasites to other members of the family.

Intestinal parasitosis was found more common in children not following the hand washing practice than those following such practice. The finding concurs with the results of Gyawali et al. (Nepal) [19], Gelaw et al. (Ethiopia) [27] and Daryani et al. (Iran) [28]. The rate of infection was significantly higher (29.4%) in children using unboiled (direct tap) water for drinking purpose whereas lower rate (9.0%) was found in children using boiled water for drinking. This pattern of infection has also been reported by Wani et al. from India [29]. The explication is that boiling of water for drinking purposes kills the microorganisms and prevents transmission of infection. Thus poor hygiene practices associated with type of water may be probable risk factors for increased parasitic infection among children.

Abdominal discomfort was the most common complaint in most of the microbiologically proven cases. It should therefore be taken as a valuable pointer for clinical suspicion of parasitic infection in this age group. Even in our study gastrointestinal symptoms were significantly found in parasites positive children (21.8%). Similar results of higher prevalence of intestinal parasitosis with abdominal discomfort was also reported by Khadka et al. [16], Shrestha et al. [18], and Gyawali et al. [19] from different parts of Nepal.

Child malnutrition still exists at alarmingly high level in countries like Nepal. Large number of people below the poverty line, lack of nutritional education, inadequate health services, lack of clean drinking water and proper sanitation all contribute to the child mortality rate. A significant association was also seen with the socioeconomic status, the prevalence rate being higher amongst the lower and lower-middle economic classes. This can be attributed to their inaccessibility to safe drinking water, unhygienic personal habits due to lack of knowledge and awareness and also indirectly to their occupation as farmers.

## Conclusion

Intestinal parasitic infection is an important public health problem in Nepal. The present study reveals that intestinal parasites are abundant among school children of Lalitpur district of Nepal. This situation strongly calls for the institution of control measures, including treatment of infected individuals, improvement of sanitation practices, and provision of safe drinking water. Poverty, lack of awareness, failure to practice proper hand washing after defecation, unsafe drinking water are some of the predisposing factors highlighted by this study as causes of parasitic infections. Appropriate health education should be given to children and their parents concerning disease transmission, personal hygiene and safe drinking water.

## Competing interests

The authors declare that they have no competing interests concerning the work reported in this paper.

## Authors’ contributions

ST, NA, AS participated in the sample collection. ST, SA, NA and AS carried out the different laboratory tests in the study. ST, BS and SG performed the statistical analysis. ST, SA, NA, AS, JG, DR, HPN and JBS conceived the design of the study and guided to draft the manuscript. HPN corrected the language of manuscript. All authors read and approved the final draft of the manuscript.
